# Rethinking Schizophrenia Care in Europe: A Brain Health Approach to Early Intervention and System Reform

**DOI:** 10.1192/j.eurpsy.2026.10517

**Published:** 2026-07-31

**Authors:** P. Mohr, G. Dom, A. Fiorillo, P. Falkai, C. Arango, A. Szulc, J. Samochowiec, P. Toczyski, J. Rybakowski, M. Nordentoft, M. Lorenzen, W. Gaebel, A. Meyer-Lindenberg, A. Vita, I. Bitter, S. Galderisi, J. Beezhold, P. Boyer, P. Keri, A. Catalan, B. Chaumette, S. Dolffus, C. Duarte, P. Fusar-Poli, O. Howes, J. Vasco Santos, V. Quoidbach

**Affiliations:** 1 National Institute of Mental Health; 2 Third Faculty of Medicine, Charles University, Prague, Czech Republic; 3 Collaborative AntwerpPsychiatric Research Institute, Antwerpen, Belgium; 4 Department of Psychiatry University of Campania “Luigi Vanvitelli”, Naples, Italy; 5 Department of Psychiatry and Psychotherapy, Munich University Hospital, Munich, Germany; 6 Child and Adolescent Department, Institute of Psychiatry and Mental Health, Hospital General Universitario Gregorio Marañón, CIBERSAM, IiSGM, School of Medicine, Universidad Complutense, Madrid, Spain; 7 Medical University of Warsaw. Department of Psychiatry, Warsaw; 8 Department of Psychiatry, Pomeranian Medical University, Szczecin; 9 Maria Grzegorzewska University (APS), Warsaw; 10 Department of Adult Psychiatry, Poznan University of Medical Sciences, Poznan, Poland; 11 Department of Clinical Medicine, University of Copenhagen; 12 Hjernerådet - The Danish Brain Council, Copenhagen, Denmark; 13 LVR-Klinikum, Department of Psychiatry and Psychotherapy, Medical Faculty, Heinrich-Heine-University, Dusseldorf; 14 Central Institute of Mental Health, Heidelberg University/Medical Faculty Mannheim, Heidelberg, Germany; 15 University of Brescia, School of Medicine, Brescia, Poland; 16 Department of Psychiatry and Psychotherapy, Semmelweis University, Budapest, Hungary; 17 Department of Mental and Physical Health and Preventive Medicine, University of Campania “Luigi Vanvitelli”, Naples, Italy; 18 Great Yarmouth Acute Service, Norfolk and Suffolk NHS Foundation Trust, Northgate Hospital, Great Yarmouth, United Kingdom; 19 European Brain Foundation; 20 GAMIAN-Europe , Brussels, Belgium; 21 Department of Neuroscience, University of the Basque Country UPV/EHU, Bilbao, Spain; 22 Université Paris Cité, Institute of Psychiatry and Neuroscience of Paris (IPNP), INSERM U1266, GHU-Paris Psychiatrie et Neursociences, Hôpital Sainte Anne, Paris; 23 University Caen Normandie, Caen, France; 24 European Federation of Psychologists’ Associations, Standing Committee on Clinical Neuropsychology, Brussels, Belgium; 25 Department of Psychosis Studies, Institute of Psychiatry, Psychology and Neuroscience, King’s College London; 26 Institute of Psychiatry, Psychology and Neuroscience, King’s College London, London, United Kingdom; 27 Department of Community Medicine, Information and Health Decision Sciences, Faculty of Medicine, University of Porto, Porto, Portugal; 28 European Brain Council, Brussels, Belgium; 29 Warwick Medical School, University of Warwick, Coventry, United Kingdom

## Abstract

**Introduction:**

Schizophrenia continues to represent a major public health concern, affecting individuals, families, and health systems throughout Europe. While progress has been made in managing positive symptoms, significant challenges persist—particularly in early detection, continuity of care, and addressing cognitive and negative symptoms. Moreover, traditional care models often neglect the critical role of social determinants and environmental factors that contribute to disease onset and progression.

**Objectives:**

This project aims to introduce a redefined care framework for schizophrenia, based on the European Brain Council’s Rethinking Schizophrenia initiative and Study Paper released in 2025. The objective is to advocate for a shift from reactive, crisis-driven care models toward a proactive, brain health–oriented paradigm that integrates clinical, social, and policy components. The project also seeks to bridge the gap between neuroscience and real-world service delivery through national “country profiles” and policy engagement.

**Methods:**

The project builds on a multi-phase methodology. Phase 1 and Phase 2 included semi-structured interviews and qualitative research across nine countries to understand patient pathways and barriers to care. Phase 3 focuses on country-specific analyses in Poland, Denmark, and Germany using a structured template. It integrates national health data, WHO and EU resources, and consultations with patients, experts and key national stakeholders to assess care delivery, social determinants, and innovation uptake as well to refine findings and recommendations.

**Results:**

Findings from Poland, Denmark, and Germany reveal persistent systemic gaps in early intervention, insufficient youth-centered services, fragmented care delivery, and limited focus on negative and cognitive symptoms. The results emphasize the need to 1) Integrate brain health principles into national policy; 2) Strengthen early neuropsychological assessment; 3) Expand access to innovative treatments and psychosocial care; 4) Address socio-environmental risk factors including poverty, trauma, and housing instability. These national profiles aim to create momentum for systemic reform.

**Conclusions:**

This project calls for a paradigm shift in schizophrenia care—from siloed mental health responses to integrated, brain health–oriented strategies that span clinical care, social policy, and prevention. Country-specific insights demonstrate the urgent need for upstream interventions, personalized care pathways, and cross-sector coordination. By placing brain health and the social determinants of health at the core of system reform, the *Rethinking Schizophrenia* project supports a holistic and sustainable vision for schizophrenia care in Europe. The project further underscores the importance of continuous EU and national policy engagement and aims to contribute to a broader “brain health in all policies” agenda.

**Disclosure of Interest:**

None Declared

